# BioActivities of *Coturnix japonica* (quail) egg yolk and albumen against physiological stress

**DOI:** 10.1002/fsn3.397

**Published:** 2016-06-18

**Authors:** Gideon O. Oladipo, Emmanuel O. Ibukun

**Affiliations:** ^1^ Department of BiochemistryFederal University of Technology AkureAkureOndo StateNigeria

**Keywords:** Antistress, cold‐restraint stress, hepatopathy, immobilization stress, quail egg yolk and albumen

## Abstract

Cold and immobilization stressors can generate oxidative stress as well as skeletal muscle fatigue. Free radicals cause oxidative degradation of lipids, proteins, nucleic acids, and carbohydrates molecules thereby compromising cell integrity and function. *Coturnix japonica* (quail) egg had been described as being very functional biochemically, due to the essential biomolecules it contains in very regulated quantity. This study was designed to evaluate the in vitro antioxidant activity of extracts of quail egg yolk and the albumen. The assessment of the antioxidant potentials was typified using the total antioxidant capacity, and ABTS, DPPH (1‐diphenyl‐2‐picrylhydrazyl) and hydroxyl radicals scavenging activities. Others are reducing power, metal chelating and lipid peroxidation inhibition activities. The antistress activities of quail egg yolk and albumen were evaluated on hepatopathic enzymes as well as endogenous antioxidant enzymes. The total antioxidant activities of the yolk extract (YE) and the albumen extracts (AE) were, respectively, 186.57 ± 6.441 mg/g and 172 ± 10.690 mg/g AAE (Ascorbic Acid Equivalent). The YE exhibited significant, potent and appreciable antioxidant activities than AE in a concentration‐dependent manner. The study confirmed that quail egg yolk contained highly antioxidative bioactive compounds not present in albumen, contributing to its (yolk) overall antioxidant and anti‐inflammatory (antistress) properties, thus necessitating their (albumen and yolk) beneficial effects in the management of oxidative and inflammatory conditions.

## Introduction

A vast amount of research has been conducted to understand the intricate cascade of events that occur once the brain detects a disruption in homeostasis (a stressor) and the hormonal responses driven by these systems (Thomas and Lena 2010; Kyrou and Tsigos 2009; Charmandari and Tsigos 2005). The key components of the “stress system” are the hypothalamic‐pituitary‐adrenal (HPA) axis and the sympathetic nervous system (SNS). When the hypothalamus is triggered by a stressor, corticotropin‐releasing hormone (CRH or CRF, corticotropin‐releasing factor), and arginine vasopressin (AVP) are secreted, eliciting both the production of adrenocorticotropin hormone (ACTH) from the posterior pituitary and the activation of the noradrenergic neurons of the locus caeruleas/norepinepherine (LC/NE) system in the brain. The LC/NE system is primarily responsible for the immediate “fight or flight” response driven by epinephrine and norepinephrine, whereas ACTH drives the production of cortisol from the adrenal cortex. Under normal conditions, the production of CRH and ACTH fluctuate in a predictable circadian cycle and are inhibited by high levels of blood cortisol via a well‐described negative feedback loop Experimental and clinical evaluations are specific for a wide range of body changes also called adaptation syndrome, which are predictable rhythm and responses of the HPA axis (Thomas and Lena 2010).

Cold and immobilization stressors can generate oxidative stress as well as skeletal muscle fatigue. Cold immobilization stress also called immobilization stress and cold‐restraint stress had been described by Popovic et al. (2009) as experimental induction of very extreme condition which cannot be carried out on human, but believed to have same effect on human as it revealed in animal models. Free radicals cause oxidative degradation of lipids, proteins, nucleic acids, and carbohydrates molecules thereby compromising cell integrity and function. The large generation of free radicals, particularly reactive oxygen species and their high activity play important roles in the progression of a great number of pathological disorders like inflammation, atherosclerosis, stroke, heart disease, diabetes mellitus, multiple sclerosis, cancer, Parkinson's, and Alzheimer's diseases (Aina and Oyedapo 2013; Ozgen et al. 2006; Mensor et al. 2001).

Several studies had predicted quail egg as medically relevant in elevating antioxidant status of experimental rats. However, this study was aimed to evaluate the antistress properties of quail egg yolk and albumen on hepatic function as well as modulation of the antioxidant status of the animals, the toxicological evaluation of diazepam used as standard antistress drug on antioxidant modulation was established.

## Methodology

Reagents and Chemicals used in this experiment were obtained from different sources such as British Drug House (BDH) and Sigma Aldrich limited and were all of good analytical grades. All the solutions, buffers, and reagents were prepared using glass distilled water. Diazepam was used in this experiment as standard antistress drug (reference drug) was obtained from Martadol pharmaceutical shop, Akure, Ondo State. Nigeria and was NAFDAC registered.

Extraction of bioactive components involved (Jianping et al.^2011^); approximately 100 g of freeze‐dried egg yolk was extracted with 500 mL of 80% methanol (80:20, vol/vol) adjusted to pH 1.5 with 1 mol/L HCl. Albumen extraction was done by enzymatic hydrolysis using pepsin (Davalos et al. 2004). The samples were then mixed thoroughly using a vortex mixer for 2 min and centrifuged at 6000*g* for 10 min at 4°C. The supernatant was collected, freeze‐dried, and reconstituted to 40 mL.

### In Vitro antioxidant assays

Since the determination of antioxidant activities is a quantitative analysis, all assays were performed in triplicate. For each test performed, various concentrations (1–5 mg/mL) of the extract and standards were prepared.

### Assay for total antioxidant activity

To the reagent solution; sulfuric acid (0.6 mol/L), sodium phosphate (28 mmol/L), and ammonium molybdate (4 mmol/L); 0.3 mL of sample was added and incubated at 95^°^C in a water bath for 90 min. After cooling to room temperature absorbance was recorded at 765 nm against reagent blank. The absorbance of the sample was extrapolated on the ascorbic acid standard curve to obtain concentration of the sample in mg/ml then the total antioxidant activity (mg/g ascorbic acid equivalence) was calculated.

### Assay of reducing power activity

The reducing power of the extracts was determined according to the method of Oyaizu (1986). Extract (0.5 mL) was mixed with 1.25 mL each of phosphate buffer and potassium ferricyanide (C_6_N_6_FeK_3_). The mixture was incubated at 50°C for 20 min. Trichloroacetic acid (1.25 mL) was then added and the mixture centrifuged at 3000 g for 10 min. Thereafter, 1.25 mL of the upper layer of the solution was mixed with 1.25 mL of distilled water and 0.25 mL of FeCl_3_. The absorbance was read at 700 nm. Higher absorbance of the reaction mixture indicates greater reductive potential.

### Assay of DPPH radical scavenging (1, 1‐diphenyl‐2‐picrylhydrazyl) activity

The DPPH radical scavenging activity of the extracts was evaluated according to the method described by Leong and Shui (2002). Exactly 1 mL of 0.3 mmol/L DPPH prepared in methanol was added to 1 mL of extract of various concentrations (1–5 mg/mL) and allowed to react at room temperature for 30 min. The reaction mixture was vortexed thoroughly and left in the dark at room temperature for 30 min. The absorbance of the mixture was measured spectrophometrically at 517 nm and the percentage scavenging activity was calculated using the formula below.

% scavenging activity = ((Ac – As)/Ac) × 100

where Ac is the absorbance of control and As the absorbance of the extract.

### Assay of 2,2'‐azino‐bis‐(3‐ethylbenzothiazoline‐6‐sulfonic acid) (ABTS) radical scavenging activity

ABTS radical scavenging activity of the plant extract was determined according to the method of Re et al. (1999). The stock solutions were of 7 mmol/L ABTS^+^ and 2.4 mmol/L potassium persulfate. The working solution was then prepared by mixing the two stock solutions in equal quantities and allowing them to react for 16 h at room temperature in the dark. The solution was diluted by mixing 5 mL ABTS^+^ solution with 145 mL of distilled water to obtain an absorbance of 0.076 ± 0.001 units at 734 nm. Extracts (1 mL) at various concentrations (1–5 mg/mL) were allowed to react with 1 mL of ABTS^+^ solution, and the absorbance was measured at 734 nm after 30 min using a spectrophotometer and the percentage scavenging activity was calculated using the formula below.

% scavenging activity = ((Ac–As)/Ac) × 100

where Ac is the absorbance of control and As the absorbance of the extract.

### Assay of metal chelating activity

The metal chelating activity was determined according to the method of Haro‐Vicente et al. (2006). Extract (1 mL) was added to 100 *μ*L of 1 mmol/L FeSO_4_.7H_2_O. The reaction mixture was left at room temperature for 2 min. After which 0.5 mL of 0.5 mmol/L 1, 10‐phenanthroline was added and the mixture was incubated for 10 min at room temperature. The absorbance was read at 510 nm. The Fe^2+^ chelating capacity was calculated thus:

Fe^2+^ chelating activity (%) = ((Ac–As)/Ac) × 100

### Assay of hydroxyl radical scavenging activity

The hydroxyl radical scavenging activity was measured by studying the competition between deoxyribose and the extracts for hydroxyl radicals generated from the Fe^2+^ /ascorbate/EDTA/H_2_O_2_ system according to the method of Halliwell et al. (1987). The reaction mixture contained 1 mL (3.0 mmol/L deoxyribose, 0.1 mmol/L EDTA, 2 mmol/L H_2_O_2_, 0.1 mmol/L l‐Ascorbic acid, 0.1 mmol/L FeCl_3_.6H_2_O in 10 mmol/L phosphate buffer, pH 7.4) and various concentrations of the extracts (1–5 mg/mL). The reaction mixtures were incubated at 37°C for 1 h, followed by the addition of 1 mL of 1 % (w/v) TBA (in 0.25 N HCl) and 1.0 mL 10 % (w/v) TCA. The reaction mixtures were heated in boiling water bath at 100°C for 20 min and the pink chromogen (malondialdehyde‐(TBA) adduct) was extracted into 1.0 mL of butan‐1‐ol and the absorbance was read at 532 nm against reagent blank. The hydroxyl radical scavenging activity calculated thus: Hydroxyl radical (OH^−^) scavenging activity (%) = ((Ac–As)/Ac) × 100.

### Assay of lipid peroxidation inhibition activity

The inhibition of lipid peroxidation was ascertained according to the method of Ohkawa et al. (1979). Albino rats were killed by cervical dislocation. The liver was carefully excised and washed in ice cold 1.15% potassium chloride solution, blotted with filter paper and weighed. The liver homogenized in four volumes phosphate buffer (pH 7.4) using a Teflon homogenizer. The resulting homogenate was centrifuged at 3000 rpm for 10 min to obtain the supernatant. A 200 *μ*L aliquot of each of the supernatant was mixed with 60 *μ*L 0.1 mol/L Tris HCl buffer (pH 7.4) and extracts of various concentrations (1–5 mg/mL) followed by the addition of 50 mmol/L FeSO_4_ and 240 *μ*L of distilled water. The resultant mixture were incubated at 37^°^C for 1 h. 600 *μ*L of 8.1% SDS was added followed by the addition of 1200 *μ*L of 1.3 mol/L Acetic acid buffer (pH 3.4) and 1200 *μ*L 0.8 % TBA. The mixture was heated at 100^°^C for 1 h to complete the reaction. Then the samples were cooled, centrifuged at 3000 rpm for 10 min. The intensity of pink colored complex was measured at 532 nm and the inhibition of lipid peroxidation was calculated thus:

Inhibition (%) = ((Ac–As)/Ac) × 100

## Experimental Design

### Immobilization Stress combined with cold‐restraint stress

Male albino rats were used according to the standard guidelines of the Care and Use of Experimental Animal Resources. 40 male albino rats weighing 200 ± 10 g were used to evaluate the ability of the quail egg yolk and albumen extracts to combat stress and were obtained from standard animal house. The rats were housed 5 per cage under constant environmental conditions (20–24°C; 12 h light/dark cycle), and were given ad libitum access to standard pelleted food and water. After the administration of quail egg yolk and albumen extracts for 21 days, combined IS and cold‐restraint stress (CRS) test was performed by immobilizing animals in the cold chamber at 4 ± 0.3°C; the plexiglass cage volume was adjusted to the size of the animal, to restrain completely their movements (Popovic et al. 2009) for 2 h, except group 1. Group 2 animals were treated after stress with diazepam (5 and 10 mg/mL BW). Immobilization was done in a plastic container with the aid of paper glue.

Group 1: Untreated, unstressed group‐Negative control group (C−).

Group 2: Untreated, stressed group‐Positive control group (C+).

Group 3: Diazepam treated (5.0 mg/mL/BW/0.2 mL ip), stressed group (D1).

Group 4: Diazepam treated (2.5 mg/mL/BW/0.2 mL ip), stressed group (D2).

Group 5: Treated (albumen 5 mg/mL/BW/0.5 mL orally), stressed group (A1).

Group 6: Treated (albumen10 mg/mL/BW/0.5 mL orally), stressed group (A2).

Group 7: Treated (yolk 5 mg/mL/BW/0.5 mL orally), stressed group (Y1).

Group 8: Treated (yolk 10 mg/mL/BW/0.5 mL orally), stressed group (Y2).

### Preparation of serum and tissue homogenates

Blood samples were collected by ocular punctures into plain bottles. Serum was prepared by aspiration of the clear liquid after clotting and centrifuged for 10 min at 3000 g in a bench centrifuge. Rats were anaesthetized by cervical dislocation and killed loss of consciousness. The liver and kidneys were excised, washed in ice cold 1.15% potassium chloride solution, blotted with filter paper and weighed, and were homogenized in ice cold 5 % w/v sodium phosphate buffer (pH 7.4) using a Teflon homogenizer. The resulting homogenates were stored at 4°C and then used for biochemical analysis.

### In vivo Biochemical Estimation

#### Liver function evaluations

##### Assay of determination of TP in liver tissues

This was carried out using the manufacturer protocol of Randox laboratory limited, United Kingdom. Total Protein Kit based on Weichselbaumin, Randox laboratory limited, United Kingdom (1995). 1 mL of reagent R1(Sodium hydroxide (100 mmol/L), sodium‐potassium tartrate (16 mmol/L), potassium iodide (15 mmol/L), and copper II sulfate [6 mmol/L]) was added to 0.02 mL of the test sample, the mixture was incubated at 25°C and the absorbance was then measured against the reagent blank at a wavelength of 546 nm.

Total Protein Concentration = ((Abs Sample/ Abs Standard) x standard concentration.

##### Assay of aspartate amino transferase activity in serum

Activity of AST was evaluated using manufacturer protocol of Randox AST Kit based on the principle of Reitman and Frankel (1957). Diluted sample (0.1 mL) was mixed with 0.5 mL of R1 (phosphate buffer (100 mmol/L, pH 7.4), l‐aspartate (100 mmol/L), and *α*‐oxoglutarate [2 mmol/L]) and the mixture incubated for 30 min at 37°C after which 0.5 mL of R2 (2, 4‐dinitrophenylhydrazine (2 mmol/L]) was added to the reaction mixture and allowed to stand for another 20 min at 25 ^0^ C. Then, 5.0 mL of NaOH (0.4 mol/L) was added and the absorbance was read against the reagent blank after 5 min at 546 nm. The activity of AST in homogenate was obtained following the extrapolation of absorbance value on AST standard curve.

##### Assay of alanine amino transferase activity in serum

Assay of alanine amino transferase (ALT) activity was carried out using the manufacturer protocol of Randox ALT Kit based on the principle described by Reitman and Frankel (1957). Reagent1 (0.5 mL) containing Phosphate buffer (100 mmol/L, pH 7.4), l‐alanine [200 mmol/L), and *α*‐oxoglutarate (2.0 mol/L) was added to a test tube already containing 0.1 mL of serum sample and the mixture was incubated at 37°C for 30 min. Then, 0.5 mL of R2 containing 2, 4‐dinitrophenylhydrazine (2.0 mmol/L) was added and the mixture incubated again at 20°C for 20 min. Finally, 5 mL of NaOH was added. The mixture was allowed to stand for 5 min at room temperature and the absorbance was read at 546 nm. The activity of ALT in the homogenate was obtained from a standard curve.

##### Assay of alkaline phosphatase activity in serum

Assay of alkaline phosphatase (ALP) activity was carried out according to the procedure provided by Randox Kit Manufacturer which is based on the method of Englehardt (1970). ALP activity was measured by monitoring the concentration of p‐nitrophenol formed when ALP reacted with p‐nitrophenyl phosphate. Exactly 1.0 mL of the reagent (1 mol/L, pH 9.8 Diethanolamine buffer, 0.5 mmol/L MgCl_2_; substrate: 10 mmol/L p‐nitrophenolphosphate) was added to 0.02 mL of sample and then mixed. The absorbance was read for 3 min at intervals of 1 min at a wavelength of 405 nm.

###### Calculation

ALP activity was determined using the formula:

U/l = 2760 × A405 nm/min.

##### Assay of determination of bilirubin concentration in serum

The colorimetric method based on that described in Randox kit protocol. Total bilirubin is determined in the presence of caffeine, which releases albumin bound bilirubin, by the reaction with diazotized sulfanilic acid, absorbance was read at 578 nm.

### Evaluation of GGT activity in serum

The assay was conducted using a Randox kit protocol. Using a water‐bathe set at 37°C for the incubation of the reaction, the reaction is constituted of 100 *μ*L of the sample extract 1000 *μ*L of the reagent at a pH of 8.25 using tris‐buffer of 100 mmol/L concentration. The reagent contains glycylglycine (100 mmol/L) and L‐*γ*‐glutamyl‐3‐carboxy‐4‐nitroanilide (2.9 mmol/L). The absorbance of the mixture was read at 405 nm.

#### Calculation

To calculate the GGT activity use the following formula.

U/L = 1158 × A 405 nm/min.

### Evaluation of endogenous antioxidant enzyme in serum

#### Superoxide dismutase activity

SOD activity was assayed by the method of Kakkar et al. (1984). Reaction mixture contained 1.2 mL of sodium pyrophosphate buffer (0.052 mmol/L, pH 7.0), 0.1 mL of phenazine methosulfate (PMS) (186 *μ*mol/L), 0.3 mL of nitro blue tetrazolium (NBT) (300 *μ*mol/L). 0.2 mL of the supernatant obtained after centrifugation (1500*g*, 10 min followed by 10,000*g*, 15 min) of 10% kidney homogenate was added to reaction mixture. Enzyme reaction was initiated by adding 0.2 mL of NADH (780 *μ*mol/L) and stopped precisely after 1 min by adding 1 mL of glacial acetic acid. Amount of chromogen formed was measured by recording color intensity at 560 nm. Results are expressed as units/mg protein.

#### Glutathione peroxidase activity (GSH‐Px)

Glutathione peroxidase (GSH‐Px) activity was measured by NADPH oxidation, using a coupled reaction system consisting of glutathione, glutathione reductase, and cumene hydroperoxide (Tappel 1978). 100 *μ*L of enzyme sample was incubated for 5 min with 1.55 mL stock solution (prepared in 50 mmol/L Tris buffer, pH 7.6 with 0.1 mmol/L EDTA) containing 0.25 mmol/L GSH, 0.12 mmol/L NADPH, and 1 unit glutathione reductase. The reaction was initiated by adding 50 *μ*L of cumene hydroperoxide (1 mg/mL), and the rate of disappearance of NADPH with time was determined by monitoring absorbance at 340 nm. One unit of enzyme activity is defined as the amount of enzyme that transforms 1 *μ*mol of NADPH to NADP per min. Results are expressed as units/mg protein.

#### Catalase activity

The activity of Catalase activity (CAT) was measured using its perioxidatic function according to the method of Johansson and Borg (1988). 50 *μ*L potassium phosphate buffer (250 mmol/L, pH 7.0) was incubated with 50 *μ*L methanol and 10 *μ*L hydrogen peroxide (0.27%). The reaction was initiated by addition of 100 *μ*L of enzyme sample with continuous shaking at room temperature (20°C). After 20 min, reaction was terminated by addition of 50 *μ*L of 7.8 mol/L potassium hydroxide. 100 *μ*L of purpald (4‐Amino‐3‐hydrazino‐5‐mercapto‐1,2,4‐triazole, 34.2 mmol/L in 480 mmol/L HCl) was immediately added, and the mixture was again incubated for 10 min at 20°C with continuous shaking. Potassium peroxidate (50 *μ*L 65.2 mmol/L) was added to obtain a colored compound. The absorbance was read at 550 nm in a spectrophotometer. Results are expressed as micromoles of formaldehyde produced/mg protein.

#### Reduced glutathione (GSH)

Reduced glutathione (GSH) level in the liver was assayed following the method of Ellman (1959), modified by Hissin and Hilf (1973). The homogenate (720 *μ*L) was double diluted and 5% TCA was added to it to precipitate the protein content of the homogenate. After centrifugation (10,000*g* for 5 min) at 4°C the supernatant was taken, 5,5'‐dithiolbis‐2‐nitrobenzoic acid (DTNB) solution (Ellmam's reagent) was added to it and the absorbance was measured at 412 nm on a spectrophotometer. A standard graph was drawn using different concentrations of standard GSH solution (1 mg/mL). With the help of the standard graph, GSH contents in the homogenates of the experimental animals were calculated.

#### Glutathione‐S‐transferase

Glutathione‐S‐transferase (GST) catalyzes the conjugation reaction with glutathione in the first step of mercapturic acid synthesis. GST activity was measured by the method of Habig and Jakoby (1974). The reaction mixture contained suitable amount of the enzyme (25 *μ*g of protein in homogenates), 1 mL of KH_2_PO_4_ buffer, 0.2 mL of EDTA, 0.1 mL of 1‐chloro‐2,4‐dinitrobenzene (CDNB), and GSH. The reaction was carried out at 37°C and monitored spectrophotometrically by the increase in absorbance of the conjugate of GSH and CDNB at 340 nm. A blank was run in the absence of the enzyme. One unit of GST activity is 1 *μ*mol product formation per minute.

All values are expressed as mean ± standard deviation. Statistical evaluation was done using One Way Analysis of Variance (ANOVA) followed by Duncan's Multiple Range Test (DMRT). The significance level was set at *P* < 0.05.

## Result and Discussion

The total antioxidant capacities of the extracts of the quail egg yolk and albumen (Table 1) were 186.57 ± 6.441 and 172.04 ± 10.690 mg/g Ascorbic acid equivalent (AAE), respectively, these results revealed that the yolk extract (YE) had higher antioxidant activity than albumen extract (AE).

**Table 1:** Total antioxidant (mg/g AAE)

Extracts	Total antioxidant (mg/g AAE)
Yolk	186.57 ± 6.441^a^
Albumen	172.04 ± 10.690^a^

Values are expressed as mean ± standard deviation (*n* = 3). Values with different superscript are significantly different (*P* < 0.05). AAE, Ascorbic Acid Equivalent.

Table 2 shows the iron reducing activities of extracts of quail egg yolk and albumen increasing in a concentration‐dependent manner. It was observed that, the iron reducing potential of YE and AE were significantly lower (*P* < 0.05) than ascorbic acid at all working concentrations (1–5 mg/mL) (*P* < 0.05). However, YE gave significantly higher ferric reducing activity than AE at all concentration (*P* < 0.05).

**Table 2:** Reducing power activities of extracts of yolk and albumen

CONC (mg/ml)	Ascorbic acid	Yolk	Albumen
1	1.37 ± 0.025^g^	0.48 ± 0.032^c^	0.2 ± 0.002^c^
2	1.85 ± 0.013^i^	0.65 ± 0.02^d^	0.21 ± 0.009^c^
3	2.31 ± 0.011^j^	0.78 ± 0.018^e^	0.20 ± 0.008^c^
4	2.38 ± 0.032^k^	0.92 ± 0.026^f^	0.36 ± 0.002^b^
5	3.26 ± 0.03^l^	1.44 ± 0.111^h^	0.39 ± 0.035^b^

Values are expressed as mean ± standard deviation (*n* = 3). Values with different superscript are significantly different (*P* < 0.05).

DPPH radical scavenging activity (Table 3) showed the proton‐donating property of the extracts at all concentrations. At 5 mg/mL the variation was significantly different; ascorbic acid (93.02 ± 0.59%) exhibited a higher radical scavenging activity than albumen (50.33 ± 2.30%) but a significantly lower activity than yolk extracts (95.61 ± 0.14%). This radical protonating activities of ascorbic acid and YE did not replicate their ferric reducing properties, however, contradicted it.

**Table 3:** DPPH radical scavenging activities of extracts of yolk and albumen

CONC (mg/ml)	Ascorbic acid	Yolk	Albumen
1	83.55 ± 1.09^e^	88.86 ± 0.27^g^	22.25 ± 0.1^a^
2	87.57 ± 1.18^f^	89.61 ± 0.23^gh^	26.27 ± 0.58^b^
3	90.63 ± 0.27^hi^	89.74 ± 0.07^gh^	36.39 ± 0.86^c^
4	91.92 ± 0.99^ij^	94.43 ± 0.08^kl^	51.19 ± 1.06^d^
5	93.02 ± 0.59^jk^	95.61 ± 0.14^l^	50.33 ± 2.3^d^

Values are expressed as mean ± standard deviation (*n* = 3). Values with different superscript are significantly different (*P* < 0.05).

The YE and AE of quail egg were fast and effective scavengers of the ABTS radical and this activity was comparable to that of trolox (Table 4). Efficiency in quenching free radicals in the system is dependent on the concentration of YE and AE, and result of Table 4 presented YE (97.15 ± 0.440%) to have significant activity than trolox (89.31 ± 0.079%) and AE (84.15 ± 0.66%) at 500 *μ*g/mL (*P* < 0.05). ABTS radical scavenging activities were concentration dependent (*P* < 0.05).

**Table 4:** 2,2'‐azino‐bis‐(3‐ethylbenzothiazoline‐6‐sulfonic acid (ABTS) radical scavenging activities of extracts of yolk and albumen

CONC (mg/ml)	Trolox	Yolk	Albumen
1	48.33 ± 0.13^a^	75.81 ± 0.43^e^	67.96 ± 0.34^b^
2	74.69 ± 0.41^d^	79.11 ± 0.87^g^	73.15 ± 0.90^c^
3	79.08 ± 0.35^g^	85.32 ± 0.95^j^	77.26 ± 0.35^f^
4	86.43 ± 0.11^k^	92.89 ± 0.47^m^	82.15 ± 0.64^h^
5	89.31 ± 0.08^l^	97.15 ± 0.44^n^	84.15 ± 0.66^i^

Values are expressed as mean ± standard deviation (*n* = 3). Values with different superscript are significantly different (*P* < 0.05).

The metal (iron) chelating activities of the extracts at different concentrations were represented in Table 5. The chelating abilities of YE and AE were found to be comparable to the reference compound (EDTA) at all working concentrations and exhibited high chelating ability which were significantly different. At 5 mg/mL concentration, YE had a lower activity (77.47 ± 0.25%) which is significantly different (*P* < 0.05) from EDTA (95.62 ± 0.16%). The albumen (35.75 ± 0.08%) had much lower activities at 5 mg/mL concentrations; inferring that its potency to chelate metals and inhibit the Fenton chemistry was lowest significantly compared to EDTA and YE.

**Table 5:** Metal chelating activities of extracts of yolk and albumen

CONC (mg/mL)	EDTA	Yolk	Albumen
1	67.25 ± 0.04^h^	35.52 ± 0.22^d^	22.91 ± 0.06^a^
2	75.5 ± 0.12^i^	47.52 ± 0.26^e^	25.81 ± 0.05^b^
3	79.67 ± 0.11^k^	56.21 ± 0.11^f^	26.07 ± 0.12^b^
4	84.35 ± 0.17^l^	64.51 ± 0.23^g^	26.56 ± 0.19^c^
5	95.62 ± 0.16^m^	77.47 ± 0.25^j^	35.75 ± 0.08^d^

Values are expressed as mean ± standard deviation (*n* = 3). Values with different superscript are significantly different (*P* < 0.05).

The hydroxyl radical scavenging activity (Table 6) represents the percentage hydroxyl ion scavenging activities of the YE and AE at varying concentrations (1–5 mg/mL). It was revealed that the concentration of the reference compound‐mannitol tested (at 5 mg/mL), significantly scavenged hydroxyl radical more than AE (91.86 ± 0.77%), no significant difference existed between the activity of the YE (97.81 ± 0.06) and mannitol (97.51 ± 0.154) (*P* < 0.05).

**Table 6:** Hydroxyl radical scavenging activities of extracts of yolk and albumen

CONC (mg/ml)	Mannitol	Yolk	Albumen
1	68.22 ± 0.08^d^	53.21 ± 0.02^c^	32.57 ± 0.18^a^
2	76.37 ± 0.06^f^	74.69 ± 0.07^e^	46.46 ± 0.36^b^
3	92.26 ± 0.12^h^	94.32 ± 0.2^i^	87.68 ± 0.06^g^
4	95.54 ± 0.09^j^	96.51 ± 0.07^k^	94.86 ± 1.39^ij^
5	97.51 ± 0.15^l^	97.81 ± 0.06^l^	91.86 ± 0.77^h^

Values are expressed as mean ± standard deviation (*n* = 3). Values with different superscript are significantly different (*P* < 0.05).

The lipid peroxidation inhibition activity (Table 7) was peculiar with the YE and AE. YE and AE had lipid peroxidation inhibition activities that were significantly lower compared to quercetin, at all concentrations (*P* < 0.05).

**Table 7:** Lipid peroxidation inhibition activities of extracts of yolk and albumen

CONC (mg/ml)	Quercetin	Yolk	Albumen
1	64.75 ± 0.53^d^	57.52 ± 0.51^c^	34.89 ± 0.48^a^
2	73.48 ± 0.29^g^	65.97 ± 0.67^e^	46.19 ± 0.29^b^
3	81.59 ± 0.14^i^	73.73 ± 0.42^g^	56.97 ± 0.55^c^
4	87.32 ± 0.38^j^	78.93 ± 0.52^h^	66.59 ± 0.27^e^
5	92.25 ± 0.12^l^	88.69 ± 0.33^k^	68.86 ± 0.28^f^

Values are expressed as mean ± standard deviation (*n* = 3). Values with different superscript are significantly different (*P* < 0.05).

### Biochemical estimations

The profile of biochemical indices such as, alanine aminotransferase (ALT), aspartate aminotransferase (AST), alkaline phosphatase (ALP), Gamma‐glutamyl transferase (GGT), catalase activities, SOD, Glutathione peroxidase (GSH‐Px) activities, and reduced glutathione (GSH) level total bilirubin and total protein concentration, were used in assessing the antistress properties of the YE and AE.

Exposure to immobilization stress may lead to increment of free radical generation which may have changed liver enzyme activities, and lipid peroxidation elevation in plasma of rats. Cold restraint and immobilization stressors in rats are known to produce oxidative stress and skeletal muscle fatigue.

Acute emotional stress (immobilization or restraint, alone or in combination with cold exposure) is one form of emotional stress, that has been widely used as a technique for and physiological changes coping ability of animals in response to stress (Pearl et al.^1966^). It had been reported that rats with restraint stress demonstrated elevation in the plasma levels of ACTH and corticosterone which occur during stress response via activation of the hypothalamic‐pituitary‐adrenal (HPA) axis (Lou et al. 2008). In response to stress, ACTH is released, which acts on the adrenal cortex to stimulate the synthesis and release of cortisol (Sadock and Sadock 2003). Increased plasma cortisol influences the mobilization of stored fat and carbohydrate reserves which in turn increases blood glucose level. The increased cortisol levels are reversed by antistress agents. Previous reports had also established that chronic vitamin E administration reduces an increase in plasma corticosterone level in rats with cold stress or immobilization stress (Lu et al. 2003).

Liver damage or hepatopathy could be confirmed by elevated activities of AST, ALP, and ALT. Chida et al. (2006) reported that although all the interactions between stress and the liver are not completely understood, there appears to be a negative association between stress on liver disease progression (Chida et al. 2006). The result of this study is in agreement with Chida et al. (2006) and some previous report on the effect of stress on liver enzymes.

Table 8 revealed the effect of quail egg yolk and albumen on physiological stress on liver enzyme markers in the serum; the alterations in the activities of ALT, AST, and ALP. Pretreatment with doses of yolk (5 and 10 mg/mL/BW) and albumen (5 and 10 mg/mL/BW), and the standard drug (diazepam) (5.0 and 2.5 mg/mL/BW) in the stressed groups, significantly (*P* < 0.05) reduced the elevated liver ALP, AST, and ALT levels, which could be due to inhibition of stimulation of sympathetic nervous system by the polyphenolic and/or other antioxidants compounds. The ability of quail egg yolk and albumen to significantly ameliorate hepatopathy by reducing the activities of ALP, AST, and ALT due to the induced stress indicated anti‐stress activity which may also be due to their membrane stabilizing ability. The slight increase (though not/slightly significant) in liver ALP, AST, and ALT levels observed in the treated stressed group with 5.0 mg/mL/BW of diazepam may be due to slight toxicity to the liver of the dose of the standard drug used.

**Table 8:** Results of biochemical effects of yolk and albumen on serum

GRPS	GSH (mg/g tissue)	ALT (U/L)	TOTAL BILIRUBIN (mg/dl)	AST (U/L)	ALP (U/L)	TOTAL PROTEIN (mg/dl)	SOD (*μ*mol/min/g tissue)	GSH‐Px (*μ*mol/min/g tissue)	CATALASE (*μ*mol/min/g tissue)	GGT (U/L)	GST (*μ*mol/min/g tissue)
C‐	51.19 ± 4.01^e^	18.26 ± 4.55^a^	1.15 ± 0.08^ab^	36.37 ± 3.71^a^	34.28 ± 0.93^a^	17.05 ± 0.81^e^	149.89 ±4.34^g^	44.59 ± 2.92^e^	90.46 ± 1.83^e^	12.61 ± 0.42^a^	5.42 ± 0.79^d^
C+	12.846 ± 0.65^a^	30.25 ± 1.09^d^	3.38 ± 0.12^e^	55.97 ± 5.89^e^	68.80 ± 0.94^d^	3.31 ± 0.89^a^	38.6 ± 4.47^a^	12.00 ± 1.12^a^	26.83 ± 1.42^a^	44.83 ± 0.02^f^	0.45 ± 0.13^a^
D1	20.182 ± 1.20^b^	24.22 ± 4.26^bc^	1.36 ± 0.15^bc^	44.28 ± 1.74^cd^	36.02 ± 2.14^ab^	12.83 ± 0.30^d^	76.154 ± 5.48^c^	20.19 ± 2.52^c^	37.5 ± 3.09^b^	21.52 ± 0.82^d^	1.40 ± 0.27^b^
D2	31.124 ± 0.76^d^	21.67 ± 1.81^ab^	1.03 ± 0.19^a^	40.43 ± 1.50^abc^	35.73 ± 0.84^ab^	12.9 ± 0.33^d^	109.89 ± 8.34^f^	26.68 ± 1.52^d^	78.33 ± 1.54^d^	14.98 ± 0.62^b^	2.78 ± 0.45^c^
A1	18.11 ± 2.41^b^	26.77 ± 2.64^cd^	1.02 ± 0.05^a^	45.47 ± 3.81^d^	40.12 ± 0.93^c^	5.49 ± 0.21^b^	57.622 ± 5.27^b^	14.86 ± 1.92b	29.15 ± 1.81^a^	27.37 ± 0.82^e^	1.63 ± 0.47^a^
A2	24.702 ± 1.15^c^	24.81 ± 1.86^bc^	1.36 ± 0.03^bc^	42.32 ± 2.16^bcd^	37.8 ± 1.78^b^	8.36 ± 0.66^c^	84.686 ± 6.01^d^	19.35 ± 1.22^c^	47.09 ± 1.35^c^	21.17 ± 0.12^d^	1.63 ± 0.49^b^
Y1	23.02 ± 2.25^c^	20.93 ± 2.56^ab^	1.65 ± 0.24^d^	39.30 ± 2.03^ab^	40.35 ± 1.22^c^	4.94 ± 0.67^b^	88.634 ± 2.91^d^	20.89 ± 1.72^c^	45.29 ± 6.94^c^	20.28 ± 0.52^c^	1.67 ± 0.26^b^
Y2	28.996 ± 2.36^d^	18.95 ± 1.84^a^	1.53 ± 0.26^cd^	37.96 ± 3.18^ab^	37.16 ± 2.92^b^	8.66 ± 0.55^c^	96.694 ± 4.17^e^	24.75 ± 2.42^d^	74.48 ± 7.79^d^	14.30 ± 0.82^b^	2.74 ± 0.10^c^

Values are expressed as mean ± standard deviation (*n* = 5). Values with different superscript are significantly different (*P* < 0.05) for each assay.

ALP, Assay of alkaline phosphatase; ALT, Assay of alanine amino transferase; AST, Assay of aspartate amino transferase; GGT, gamma‐glutamyl transferase; GSH; Reduced glutathione; GST, Glutathione‐S‐transferase; SOD, Superoxide dismutase.

Gamma glutamyl transferase activity was evaluated in the serum. The untreated but stressed group (C+) revealed higher activity for GGT, confirming the denaturing effect of stress. All the treated groups demonstrated ameliorative indices, however, administration of yolk extract of 10 mg/mL/BW demonstrated more ameliorative activity than albumen and leading to the reduced production of this enzyme by hepatic cells. Elevated serum GGT can be found in patients with hepatitis, biliary, and pancreas related disorders. The primary role of cellular GGT is to metabolize extracellular reduced glutathione (GSH), allowing for precursor amino acids to be assimilated and reutilize for intracellular GSH synthesis (Lee et al. 2004). The frequent denaturation of protein in the extracellular environment by activities of free radicals promote the activities of the GGT as more amino acids are made available for transmembrane mobility leading to the generation of glutathione necessary for the endogenous antioxidant activities against the radicals. However, Lee et al. (2004) reported that the increased in the activity GGT in the presence of iron and other transition metals would lead to the generation of free radicals through the reducing ability of cysteinylglycine which reduces F^3+^ to Fe^2+^ which is one of the deleterious radical of protein, lipid, and nucleic acids. We can therefore infer that the increase in GGT ectoactivity is a compromising factor leading to the cell proliferation.

Bilirubin is a natural product of heme catabolism by heme oxygenase in the liver when worn out red blood cell is broken down often due to oxidative proliferation. Serum bilirubin is considered as one of the true test of liver function since it reflects the liver ability to take up bilirubin and metabolize it to bile. Elevated levels of total bilirubin as a result of the unconjugated form indicate the severity of the acute hepatopathy resulting from physiological stress. The serum levels of the total bilirubin by antistress effect of the extracts were shown in Table 8. From the result, all the extracts have ameliorating properties. The stressed but untreated (C+) group had higher total bilirubin which was significantly different from the unstressed and untreated group (C−). The result also expressed toxic effect of posttreatment with 5 mg/mL/BW diazepam, whereas the 2.5 mg/mL/BW had a reduced total bilirubin which is significantly different from 5 mg/mL/BW. This suggested 2.5 mg/mL/BW diazepam as better antistress dose over 5 mg/mL/BW. Yolk administered group showed higher total bilirubin than albumen and diazepam. This was due to the conjugated bilirubin which on aggregate contributed to the total bilirubin concentration. The relationship between total bilirubin and stress could be traced to be oxidative proliferation of the erythrocyte membrane leading to denaturation of hemoglobin, this further led to cascade of reaction that generated total bilirubin. Moreover, hepatopathy would cause defect in the conjugation of bilirubin, leading to increased release of unconjugated bilirubin into the blood, justifying the hepatoprotective properties of the quail egg yolk and albumen.

The contributions of the bioactive compounds in the yolk and albumen to the inhibition of denaturation could not be simplified. We assume that the antidenaturation effects of the quail egg yolk and albumen revealed their relevance as possessing the ability to prevent the proliferation of the proteins by scavenging‐free radicals generated by physiological stress. The result of the liver tissue total protein evaluation revealed that there was no significant difference between the two diazepam concentrations administered, however, they showed considerable ameliorating effects but were not able to nullify the effect of the cold immobilization stress. Yolk (10 mg/mL/BW) and albumen (10 mg/mL/BW) ameliorated stressed condition but without significant difference (*P* < 0.05).

The modulation of endogenous antioxidant biomolecules in the blood revealed that the yolk and albumen facilitated the hepatic antioxidant system from the damage induced by the stress. Cells have cooperative defense systems for the reduction in oxidizing agents. The defense systems contain numerous enzymatic and nonenzymatic antioxidants, including SOD, catalase (CAT), glutathione peroxidase (GPx), reduced glutathione (GSH), and glutathione S‐transferase (GST). However, when there is an over‐flux of free radical, protective systems are overwhelmed. Catalase catalyzes the breakdown of hydrogen peroxide which is a reactive oxygen species to water and molecular oxygen, thus minimizing the proliferation of cells. SOD is one important enzyme involved in the dismutation of superoxide radical. SOD protects against free radical injury by converting superoxide radical to hydrogen peroxide and prevents the formation of hydroxyl radical. In the stressed and untreated group (C+) there was significant decrease in the activities of SOD and catalase. During the advent of free radicals, the antioxidant system of the body is compromise. Initially, there will be increase in the activities of the enzymes. However, prolong assault will result in proliferation of the hepatocytes as well as other sites of synthesis of these enzymes, thus leading to reduction in their activities. The result revealed endogenous‐enzymes‐activity promoting ability of yolk and albumen which increased as the dose increased. Yolk demonstrated significantly higher ameliorative property than albumen. Excess free radicals have been shown to react with several amino acid residues *in vitro,* making active enzymes denatured (Stadtman and Berlett 1998). Thus, treatment with yolk and albumen had a significant reduction in free radicals influence. The bioactive compounds of the extracts act as ligands that promote the genetic transcriptional and translational production of these endogenous antioxidant enzymes increasing the antioxidant status of the experimental animals.

## Conclusion

This study revealed that the extracts of egg yolk and albumen have antioxidant activities. The yolk demonstrated higher antistress and antioxidant modulating activity than higher concentrations of diazepam and albumen. The activity of 5 mg/mL/kg BW diazepam was revealed as toxic dose. The albumen antistress property could be as a result of the free R‐group of the peptides which had been described to be responsible for the biological roles of the amino acids. This study thus established that the increased consumption of *Coturnix japonica* (quail) egg could curtail physiological stress.

## Conflict of Interest

The authors declare no conflict of interest.

## References

[fsn3397-bib-0001] Aina, O. I. , and O. O. Oyedapo . 2013 In vitro investigations into the antioxidant and anti‐Inflammatory potentials of the fractions and ethanolic extract of *Cyclosorus afer* (christ.) Ching, stalks. Life J. Sci. 15:235–249.

[fsn3397-bib-0002] Charmandari, E. , and C. Tsigos . 2005 Endocrinology of the stress response. Annu. Rev. Physiol. 67:259–284. 15709959 10.1146/annurev.physiol.67.040403.120816

[fsn3397-bib-0003] Chida, Y. , N. Sudo , and C. Kubo . 2006 Does Stress Exacerbate Liver Disease? J. Gastroenterol. Hepatol. 20:202–208. 10.1111/j.1440-1746.2006.04110.x 16460474

[fsn3397-bib-0004] Davalos, A. , M. Miguel , B. Bartolome , and R. Lopez‐Fandino . 2004 Antioxidant activity of peptides derived from egg white proteins by enzymatic hydrolysis. J. Food Prot. 9:1939–1944. 10.4315/0362-028x-67.9.1939 15453585

[fsn3397-bib-0005] Ellman, G. L. 1959 Tissue sulfhydryl group. Arch. Biochem. Biophys. 82:70–77. 13650640 10.1016/0003-9861(59)90090-6

[fsn3397-bib-0006] Englehardt, A . 1970 Measurement of alkaline phosphatase. Aerztl Labor 16:42–43.

[fsn3397-bib-0007] Habig, W. H. , and W. B. Jakoby . 1974 Glutathione Stransferases. The first enzymatic step in mercapturic acid formation. J. Biol. Chem. 249:7130–7139. 4436300

[fsn3397-bib-0008] Halliwell, B. , J. M. C. Gutteridge , and O. I. Aruoma . 1987 The deoxyribose method: a simple ‘test tube’ assay for determination of rate constants for reactions of hydroxyl radicals. Anal. Biochem. 165:215–219. 3120621 10.1016/0003-2697(87)90222-3

[fsn3397-bib-0009] Haro‐Vicente, J. F. , C. Martinez‐Gracia , and G. Ros . 2006 Optimisation of invitro measurement of available iron from different fortification in citric fruit juices. J. Food Chem. 98:639–648.

[fsn3397-bib-0010] Hissin, P. J. , and R. Hilf . 1973 A fluorometric method for the determination of oxidized and reduced glutathione in tissue. Anal. Biochem. 74:214–226. 10.1016/0003-2697(76)90326-2 962076

[fsn3397-bib-0011] Jianping, W. , L. Daise , S. Andreas , and N. Chamila . 2011 Free aromatic amino acids in egg yolk show antioxidant properties. Food Chem. 129:155–161.

[fsn3397-bib-0012] Johansson, L. H. , and L. A. Borg . 1988 A spectrophotometric method for determination of catalase activity in small tissue samples. Anal. Biochem. 174:331–336. 3064653 10.1016/0003-2697(88)90554-4

[fsn3397-bib-0013] Kakkar, P. , B. Das , and P. N. Viswanathan . 1984 A modified spectrophotometeric assay of superoxide dismutase. Ind. Biochem. Biophys. 21:130–132. 6490072

[fsn3397-bib-0014] Kyrou, I. , and C. Tsigos . 2009 Stress hormones: physiological stress and regulation of metabolism. Curr. Opin. Pharmacol. 9:787–793. 19758844 10.1016/j.coph.2009.08.007

[fsn3397-bib-0015] Lee, D. , R. Blomhoff , and D. R. Jacobs . 2004 Is serum gamma‐glutamyltransferase a marker of oxidative stress? Free Radical Res. 38:535–539. 15346644 10.1080/10715760410001694026

[fsn3397-bib-0016] Leong, L. P. , and G. Shui . 2002 An investigation of antioxidant capacity of fruits in Singapore markets. Food Chem. 76:65–75.

[fsn3397-bib-0017] Lou, L. X. , B. Geng , J. B. Du , and X. C. Tang . 2008 Hydrogen sulfide‐induced hypothermia attenuates stress‐induced ulceration in rats. J. Exp. Pharmacol. Physiol. 35:223–228. 10.1111/j.1440-1681.2007.04812.x 17941893

[fsn3397-bib-0018] Lu, P. , N. Yaras , A. Agar , S. Gümüslü , S. Bilmen , and G. Özkaya . 2003 The effect of vitamin E on stress‐induced changes in visual evoked potentials (VEPs) in rats exposed to different experimental stress models. Acta Ophthalmol. Scand. 81:181–187. 12752059 10.1034/j.1600-0420.2003.00040.x

[fsn3397-bib-0019] Mensor, L. L. , F. S. Menezes , G. G. Leitao , A. S. Reis , T. C. Dos‐Santos , C. S. Coube , et al. 2001 Screening of Brazilian plant extracts for antioxidant activity by the use of DPPH free radical method. Phytother. Res. 15:127–130. 11268111 10.1002/ptr.687

[fsn3397-bib-0020] Ohkawa, H. , N. Ohishi , and K. Yagi . 1979 Assay for lipid peroxides in animal tissues by thiobarbituric acid reaction. Anal. Biochem. 95:351–358. 36810 10.1016/0003-2697(79)90738-3

[fsn3397-bib-0021] Oyaizu, M. 1986 Studies on products of browning reactions: antioxidant activities of products of browning reaction prepared from glucose amine. Jpn. J. Nutr. 44:307–315.

[fsn3397-bib-0022] Ozgen, M. , R. N. Reese , A. Z. Tulio , J. C. Scheerens , and A. R. Miller . 2006 Modified 2, 2‐ azino‐bis‐3‐ethylbenzothiazoline‐6‐sulfonic acid (ABTS) method to measure antioxidant capacity of selected small fruits and comparison to ferric reducing antioxidant power (FRAP) and 2, 2'‐ diphenyl‐1‐picrylhydrazyl (DPPH) methods. J. Agri. Food Chem. 54:1151–1157. 10.1021/jf051960d 16478230

[fsn3397-bib-0023] Pearl, W. , T. Balazs , and D. A. Buyske . 1966 The effect of stress on serum transaminase activity in the rat. Life Sci. 5:67–74. 4379856 10.1016/0024-3205(66)90188-3

[fsn3397-bib-0024] Popovic, M. , S. J. Hudomal , B. Kaurinovic , J. Rasic , S. Trivic , and M. Vojnović . 2009 Antioxidant effects of some drugs on immobilization stress combined with cold restraint stress. Molecules 14:4505–4516. doi:10.3390/14114505. 19924083 10.3390/molecules14114505 PMC6254731

[fsn3397-bib-0025] Re, R. , N. Pellegrini , A. Proteggente , A. Pannala , M. Yang , and C. Rice‐Evans . 1999 Antioxidant activity applying an improved ABTS radical cation decolorization assay. Free Rad. Biol. Med. 26:1231–1237. 10381194 10.1016/s0891-5849(98)00315-3

[fsn3397-bib-0026] Reitman, S. , and S. A. Frankel . 1957 Colorimetric method for the determination of serum level of glutamate‐oxaloacetate and pyruvate transaminases. Am. J. Clin. Pathol. 28:56–63. 13458125 10.1093/ajcp/28.1.56

[fsn3397-bib-0027] Sadock, B. , and V. Sadock . 2003 Synopsis of psychiatry. 9thed. Williams and Wilkins, PA philadelphia.

[fsn3397-bib-0028] Stadtman, E. R. , and B. S. Berlett . 1998 Reactive oxygenmediated oxidation in aging and disease. Drug Metab. Rev. 30:225–243. 9606602 10.3109/03602539808996310

[fsn3397-bib-0029] Tappel, A. L. 1978 Glutathione peroxidase and hydroperoxides. Methods Enzymol. 52:506–513. 672654 10.1016/s0076-6879(78)52055-7

[fsn3397-bib-0030] Thomas, G. G. , and E. Lena . 2010 Chronic stress and the HPA axis: clinical assessment and therapeutic considerations. A review of natural and nutraceutical therapies for clinical practice. 9; Article 2. Available at: www.pointinstitute.org/wp-content/uploads/2012/10/standard_v_9.2_hpa_axis.pdf

[fsn3397-bib-0031] Weichselbaumin, T. E. 1995 An accurate and rapid method for the determination of protein in small amountof blood serum. Am. J. Clin. Pathol. 16:40. 21027099

